# Screening of stable resistant accessions and identification of resistance loci to *Barley yellow mosaic virus* disease

**DOI:** 10.7717/peerj.13128

**Published:** 2022-03-17

**Authors:** Yuhan Pan, Juan Zhu, Yi Hong, Mengna Zhang, Chao Lv, Baojian Guo, Huiquan Shen, Xiao Xu, Rugen Xu

**Affiliations:** 1 Yangzhou University, Key Laboratory of Plant Functional Genomics of the Ministry of Education/Jiangsu Key Laboratory of Crop Genomics and Molecular Breeding/Jiangsu Co-Innovation Center for Modern Production Technology of Grain Crops/Institutes of Agricultural Science, Yangzhou, Jiangsu, China; 2 Jiangsu Institute for Seaside Agricultural Sciences and Yancheng Academy of Agricultural Science, Yancheng, Jiangsu, China

**Keywords:** Barley (*Hordeum vulgare* L.), Barley yellow mosaic disease (BYMD), Genome-wide association study (GWAS), Additive main effects and multiplicative interaction (AMMI), Stable resistant accessions

## Abstract

**Background:**

The disease caused by *Barley yellow mosaic virus* (BaYMV) infection is a serious threat to autumn-sown barley (*Hordeum vulgare* L.) production in Europe, East Asia and Iran. Due to the rapid diversification of BaYMV strains, it is urgent to discover novel germplasm and genes to assist breeding new varieties with resistance to different BaYMV strains, thus minimizing the effect of BaYMV disease on barley cropping.

**Methods:**

A natural population consisting of 181 barley accessions with different levels of resistance to BaYMV disease was selected for field resistance identification in two separate locations (Yangzhou and Yancheng, Jiangsu Province, China). Additive main effects and multiplicative interaction (AMMI) analysis was used to identify accessions with stable resistance. Genome-wide association study (GWAS) of BaYMV disease resistance was broadly performed by combining both single nucleotide polymorphisms (SNPs) and specific molecular markers associated with the reported BaYMV disease resistance genes. Furthermore, the viral protein genome linked (VPg) sequences of the virus were amplified and analyzed to assess the differences between the BaYMV strains sourced from the different experimental sites.

**Results:**

Seven barley accessions with lower standardized Area Under the Disease Progress Steps (sAUDPS) index in every environment were identified and shown to have stable resistance to BaYMV disease in each assessed location. Apart from the reported BaYMV disease resistance genes *rym4* and *rym5*, one novel resistance locus explaining 24.21% of the phenotypic variation was identified at the Yangzhou testing site, while two other novel resistance loci that contributed 19.23% and 19.79% of the phenotypic variation were identified at the Yancheng testing site, respectively. Further analysis regarding the difference in the VPg sequence of the predominant strain of BaYMV collected from these two testing sites may explain the difference of resistance loci differentially identified under geographically distinct regions. Our research provides novel genetic resources and resistance loci for breeding barley varieties for BaMYV disease resistance.

## Introduction

Barley yellow mosaic virus disease is a soil-borne virus disease caused by a single or combined infection of *Barley yellow mosaic virus* (BaYMV) and *Barley mild mosaic virus* (BaMMV), which seriously affects the production of autumn-sown barley (*Hordeum vulgare* L.) (Chen et al., 1996; Chen et al., 1999; Jiang et al., 2020). These two viruses have produced many pathogenic strains in barley, causing 70% or greater yield loss. For instance, the BaYMV, BaYMV-2, and BaMMV strains have been reported in Europe (Meyer & Dessens, 1996; Hariri, Meyer & Prud’homme, 2003), four new BaYMV strains have been reported in Japan (Nishigawa et al., 2008), one specific BaYMV strain has been reported in South Korea (Lee et al., 2006) and a series of unique BaYMV and BaMMV strains have been reported in China (Zheng et al., 1999). The emerging diversified BaYMV strains have considerable virulence, which has brought severe challenges to breed new accessions of barley resistant to BaYMV disease (Chen, 2006).

The evaluation of BaYMV disease resistance is conducted by combining BaYMV disease symptoms in the field and assessment by enzyme-linked immunosorbent assay (ELISA) in the laboratory. In a previous report, 95 accessions that showed resistance to BaYMV-2 strain were identified (Huth, 1991). Furthermore, 123 accessions were identified as being resistant to all European BaYMV strains after screening 2,000 barley accessions through combined assessment of visual evaluation and ELISA (Habekuss et al., 2000). Among 50 accessions collected from 16 regions of South Korea, which showed resistance symptoms, only one was found to maintain high degrees of resistance to all BaYMV strains tested using ELISA (So, Lee & Chon, 1997). A similar study was carried out on 26 Korean hulless barley accessions, of which four accessions showed stable resistance to BaYMV disease (Park et al., 2009). In China, disease severity scoring has been widely conducted to identify disease resistance germplasm since the end of the 1970s. More than 10,000 local barley accessions from 29 provinces were screened in different areas by plant pathologists. A series of resistant genotypes have been identified and used to breed new accessions of barley resistant to BaMYV disease (Chen et al., 1996; Chen, 2006).

Differential expression levels of resistance genes lead to the variation in tolerance levels to BaYMV disease in different barley accessions. To date, 22 BaYMV disease resistance genes have been identified by linkage analysis or cloned by using chromosome walking (Jiang et al., 2020). Six of these resistance genes are alleles of the *eukaryotic translation initiation factor 4E* (*eIF4E*), showing resistance to specific viral strains. For instance, *rym6* and *rym10* direct resistance to BaYMV, *rym*_*HOR4224*_ directs resistance against BaMMV, while *rym*_*HOR3298*_ mediates broad-spectrum resistance to multiple strains of BaYMV and BaMMV (Perovic et al., 2014; Yang et al., 2017; Shi et al., 2019). As members of the *eIF4E* allelic family, located on the terminal region of chromosome 3H, *rym4* and *rym5* were once widely targeted and utilized in barley breeding in Europe. *rym4* and *rym5* initially provided broad-spectrum resistance until the resistance provided by *rym4* was overcome by BaYMV-2 (Habekuss et al., 2008). Moreover, *rym5* directed resistance to BaYMV-2 was subsequently overcome by two new BaMMV strains (Kanyuka et al., 2004; Habekuss et al., 2008), which downgraded the value of *rym4* and *rym5* in barley breeding programs. BaYMV adaptation to barley *eIF4E*-mediated resistance can be viewed as a two-component molecular ‘arms race’ (Yang et al., 2017), for which viral infection is directly linked to the VPg-eIF4E interaction module. BaYMV overcomes *rym4/rym5*-mediated resistance by conversion of the amino acid sequence composition of its VPg protein (Stein et al., 2005; Charron et al., 2008; Habekuss et al., 2008; Li & Shirako, 2015; Rolland et al., 2017).

Another cloned gene *rym1/11* from barley accession ‘Mokusekko 3’, which is located on chromosome 4H and encodes the *protein disulfide isomerase like 5-1* (*PDIL5-1*) protein, a protein which has been shown to assist in the translation of BaYMV derived proteins during infection, has also been identified as a BaYMV disease resistance gene. A loss-of-function mutation in *PDIL5-1* interrupted the translation process of the virus to provide the plant with broad-spectrum resistance to the virus (Yang et al., 2014b). However, the disease resistance of *rym1/11* has already been overcome by new BaYMV strains in Japan (Okada et al., 2004). In addition to the cloned *eIF4E* and *PDIL5-1* resistance alleles, *rym7* (resistance against BaMMV) (Yang et al., 2013), *Rym16*^*Hb*^ (from *Hordeum bulbosum*, resistance against all strains) (Ruge-Wehling et al., 2006; Johnston et al., 2015), *Rym17* (from barley landrace, resistance against BaYMV) (Kai et al., 2012) and *Rym14*^*Hb*^ (from *Hordeum bulbosum*, resistance against all strains) (Pidon et al., 2020) have been precisely mapped to small regions on chromosomes 1H, 2H, 3H, and 6H, respectively. The remaining ten BaYMV disease resistance genes *rym2* (resistance against several strains of the two viruses) (Gotz & Friedt, 1993), *rym3* (resistance against several BaYMV strains) (Werner et al., 2003b), *rym7t* (resistance against several BaYMV strains) (Takata et al., 2012), *rym8* (resistance against several strains of the two viruses) (Bauer et al., 1997), *rym9* (resistance against several BaMMV strains) (Werner, Friedt & Ordon, 2005), *rym12* (resistance against several strains of the two viruses) (Gotz & Friedt, 1993), *rym13* (resistance against several strains of the two viruses) (Werner et al., 2003a), *rym15* (resistance against BaMMV) (Le Gouis et al., 2004), *rym18* (resistance against BaYMV) (Kai et al., 2012), and *Un-designated* (resistance against BaYMV) (Saeki et al., 1999) have only been preliminarily mapped. The stacking of several known disease resistance genes by breeding can improve the resistance of barley accessions to BaYMV disease (Yang et al., 2017; Jiang et al., 2020). Consequently, it is necessary to discover more BaYMV disease resistance genes and evaluate their field performance to benefit both the current and future disease resistance breeding for this important cereal species (Ordon et al., 2009).

Compared with traditional linkage mapping, association analysis not only shortens gene mapping time, but also overcomes the inability to detect quantitative trait loci (QTL) with low heritability (Mackay & Powell, 2007; Burghardt, Young & Tiffin, 2017). The rapid development of high-throughput sequencing technology and high-resolution association analysis at the whole genome level is conducive to identify resistance genes, leading to high efficiency in disease resistance breeding (Yu & Buckler, 2006; Zhu et al., 2008; Mohammadi et al., 2015, Mascher et al., 2017; Zeng et al., 2018; Monat et al., 2019; Mascher et al., 2021). Therefore, genome-wide association study (GWAS) has become a well-acknowledged tool for the identification of disease resistance genes. According to incomplete data, 647 QTLs (including duplicate or redundant loci) for disease resistance to all pathogens have been reported by GWAS in barley to date utilizing a mixed linear model (MLM) analysis (Igartua, Cantalapiedra & Casas, 2019), including *Fusarium* head blight resistance loci (Massman et al., 2011; Mamo, 2015), stem rust resistance loci (Turuspekov et al., 2016) and stripe rust resistance loci (Belcher et al., 2018). Recently, GWAS was performed using the fixed and random model circulating probability unification (FarmCPU) approach to identify barley scald resistance genes (Hautsalo et al., 2021) and net blotch resistance loci (Clare et al., 2021). Compared with the traditional MLM analysis, the FarmCPU approach shortens the calculation time and improves the efficiency of QTL identification (Liu et al., 2016).

In this study, we evaluated BaYMV disease resistance of 181 barley accessions in two different sites in 2 or 3 years to identify: (1) barley accessions harboring stable resistance by AMMI analysis, (2) associations between reported major genes and BaYMV disease resistance, and (3) new QTL for BaYMV disease resistance. Our results lay a foundation for the subsequent mining of novel resistance genes and the sustainability of resistance breeding to BaYMV disease in barley.

## Materials and Methods

### Plant material

A collection of 181 barley accessions sourced from around the world were used for GWAS in this study (Table S1). All the accessions were firstly genotyped using the genotyping by sequencing (GBS) approach. Over 17,000 SNP markers were scored with the assistance of USDA-ARS/Kansas State University (Fan et al., 2017). After omitting those markers with a missing rate ≥20%, a minor allele frequency (MAF) ≤0.05 and heterozygosity ≥5%, 3,818 SNP markers with unique physical position (Based on reference genome Morex V3, Mascher et al., 2021) were obtained. Markers associated with known BaYMV disease resistance genes were collected from previous studies (Table S2), and these markers were further genotyped in these 181 accessions using a standard PCR approach (Shi et al., 2019). Afterwards, 21 gene markers with polymorphism were confirmed and added to the total molecular markers (Table S2). Therefore, a total number of 3,839 molecular markers (Tables S3 and S4) were used for linkage disequilibrium (LD) analysis and GWAS.

Infected leaves of two varieties, Supi 1 (only susceptible to BaYMV) and Dan 2 (only susceptible to BaMMV), were collected to identify the differences in sequences between BaYMV and BaMMV.

### Field experiments

All plant materials reported in this study were autumn-sown (late October) and cultivated at the BaYMV disease identification nursery (BaYMV/BaMMV-infected) and *via* the use of three biological replicates. Two nursery sites were selected in this study. One site is located in the main campus of Yangzhou University (32°23′36″ N, 119°25′36″ E) with an average rainfall of 63.76 mm and daily temperature ranging from 6 °C to 15 °C and the other one is located in Yancheng (Jiangsu Institute for Seaside Agricultural Sciences, 33°25′17″ N, 120°12′ 48″ E) with an averaged rainfall of 40.52 mm and daily temperature ranging from 4 °C to 15 °C. Disease symptoms were scored in March. The experiments in Yangzhou were conducted in 2015, 2016, and 2020 growing seasons (2015 Yangzhou, 2016 Yangzhou, and 2020 Yangzhou), and those in Yancheng were in 2019 and 2020 growing seasons (2019 Yancheng and 2020 Yancheng). All accessions were cultivated under a conventional water and fertilizer management regime.

### Visual evaluation of BaYMV disease resistance

**Figure 1:**
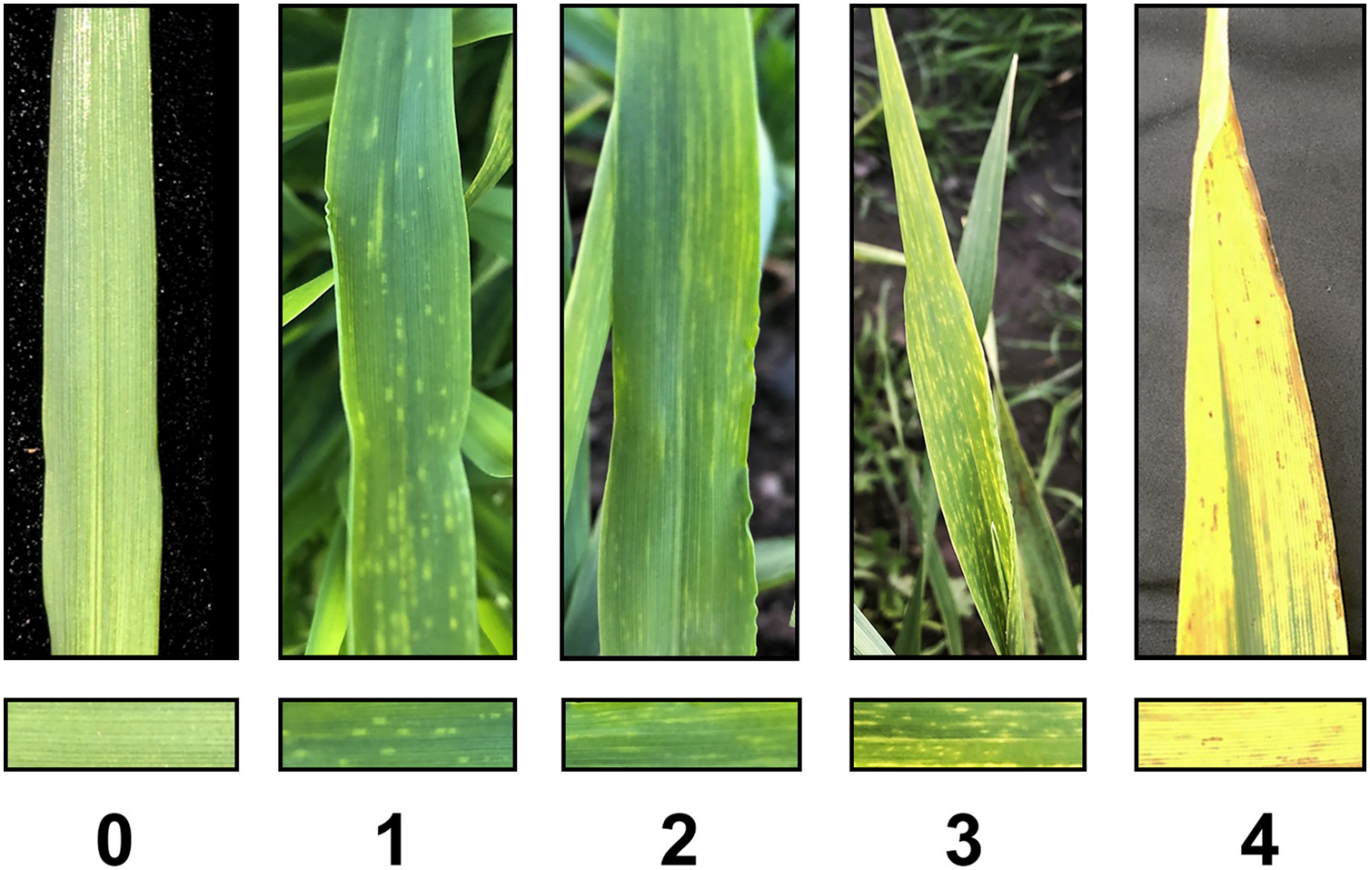
BaYMV disease grade under natural disease nursery. BaYMV disease levels were divided into different levels of severity, 0 means healthy leaves with no symptoms of disease, 1 represents typical chlorotic spots, 2 represents a quarter of the leaves displaying a yellow mosaic pattern, 3 represents more than half of the leaves displaying a yellow mosaic pattern, and 4 represents more than three-quarters of the leaves displaying a yellow mosaic pattern.

For obtaining accurate visual evaluation, disease severity scores were conducted four times within seven-day intervals. Severity scores of 0 to 4 represent the damage caused by virus infection from light to severe (Fig. 1): 0 means healthy leaves with no symptoms of disease, 1 represents typical chlorotic spots, 2 indicates a quarter of the leaves display a yellow mosaic pattern, 3 represents more than half of the leaves display a yellow mosaic pattern, and 4 indicates more than three-quarters of the leaves display yellow mosaic pattern (Pan et al., 2021). Afterwards, a standardized area under the disease progress steps (sAUDPS) index was used for the evaluation of disease resistance, which can effectively combine and transform multiple investigations of disease grades into a single value for further GWAS analysis (Simko & Piepho, 2012).

### Data analysis

Descriptive statistics, analysis of variance (ANOVA), Pearson correlation analysis, and *t*-test were analyzed using IBM SPSS Statistics 22 software. The lme4 package of R (Bates et al., 2015) was used to perform the best linear unbiased prediction (BLUP) of the sAUDPS index with repeats for different years (Fernando & Grossman, 1989). BLUPs of all the accessions were used for GWAS.

To analyze the stability of phenotypic values in different environments, the sAUDPS indices of the natural population at the Yangzhou site for 3 years and the Yancheng site for 2 years were analyzed by additive main effects and multiplicative interaction (AMMI) analysis. Firstly, principal component analysis (PCA) was used to analyze the interaction between the sAUDPS index and the environment, and the significance of each principal component was judged. If the principal components were significantly different, it means that they meet the requirements of model analysis (Crossa et al., 1991; Lei & Xi, 2006; Jiwuba et al., 2020). The AMMI analysis was analyzed by using the Agricola package in R (https://rdrr.io/cran/agricolae/man/agricolae-package.html). The equations of the model and the calculation formulas of the stability parameters are as follows:

${y_{ijk}} = {\rm \mu } + {{\rm \alpha }_i} + {{\rm \beta }_j} + \mathop \sum \limits_{r = 1}^n {{\rm \theta }_r}{{\rm \gamma }_{ir}}{{\rm \delta }_{jr}} + {{\rm \rho }_{ij}} + {{\rm \varepsilon }_{ijk}}$

$D = \sqrt {\mathop \sum \limits_{r = 1}^N {W_r}{\rm \gamma }_{{\rm i}r}^2}$

In these two formulas, $\; {y_{ijk}}$ is the trait of the *i*– accession in the *j*— environment the phenotypic value repeated for the *K* μtime, μ is the average value of the phenotype, ${{\rm \alpha }_i}$ is the genotype main effect of the *i*– accession, ${{\rm \beta }_j}$ is the environment of the *j*— environmental main effect, ${{\rm \theta }_r}$ is the *r* principal component eigenvalue under the interaction of genotype and environment, ${{\rm \gamma }_{ir}}$ is the genotype score of the *r* principal component under genotype by environment interaction, ${{\rm \delta }_{jr}}$ is the environmental score of the *r* principal component under genotype by environment interaction, ${{\rm \rho }_{ij}}$ is the residual error, and ${{\rm \varepsilon }_{ijk}}$ is the test error. The stability parameter *D value* refers to the Euclidean distance between the position of an accession in the principal component space of the interaction and the origin. ${W_r}$ is the variance explanation rate of each principal component. The lower the *D value*, the more stable the phenotypic value.

**Figure 2:**
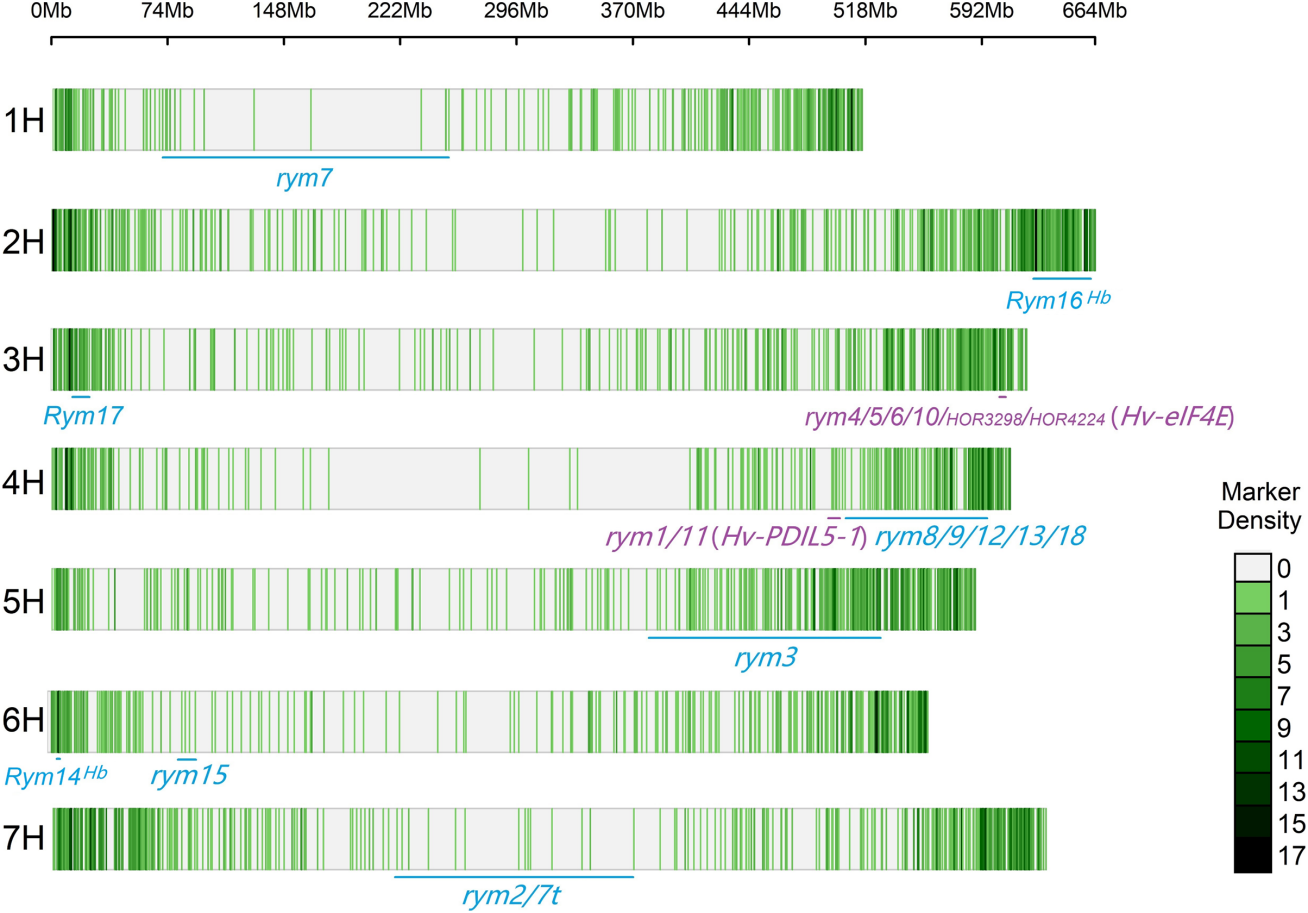
Distribution of molecular markers used in this study and known BaYMV disease resistance genes on barley chromosomes. *Rym* represents a dominant gene, while *rym* represents a recessive gene. The genes in the color blue represent genes that have been assigned a chromosome position, and genes in the color purple represent genes that have been cloned.

**Figure 3:**
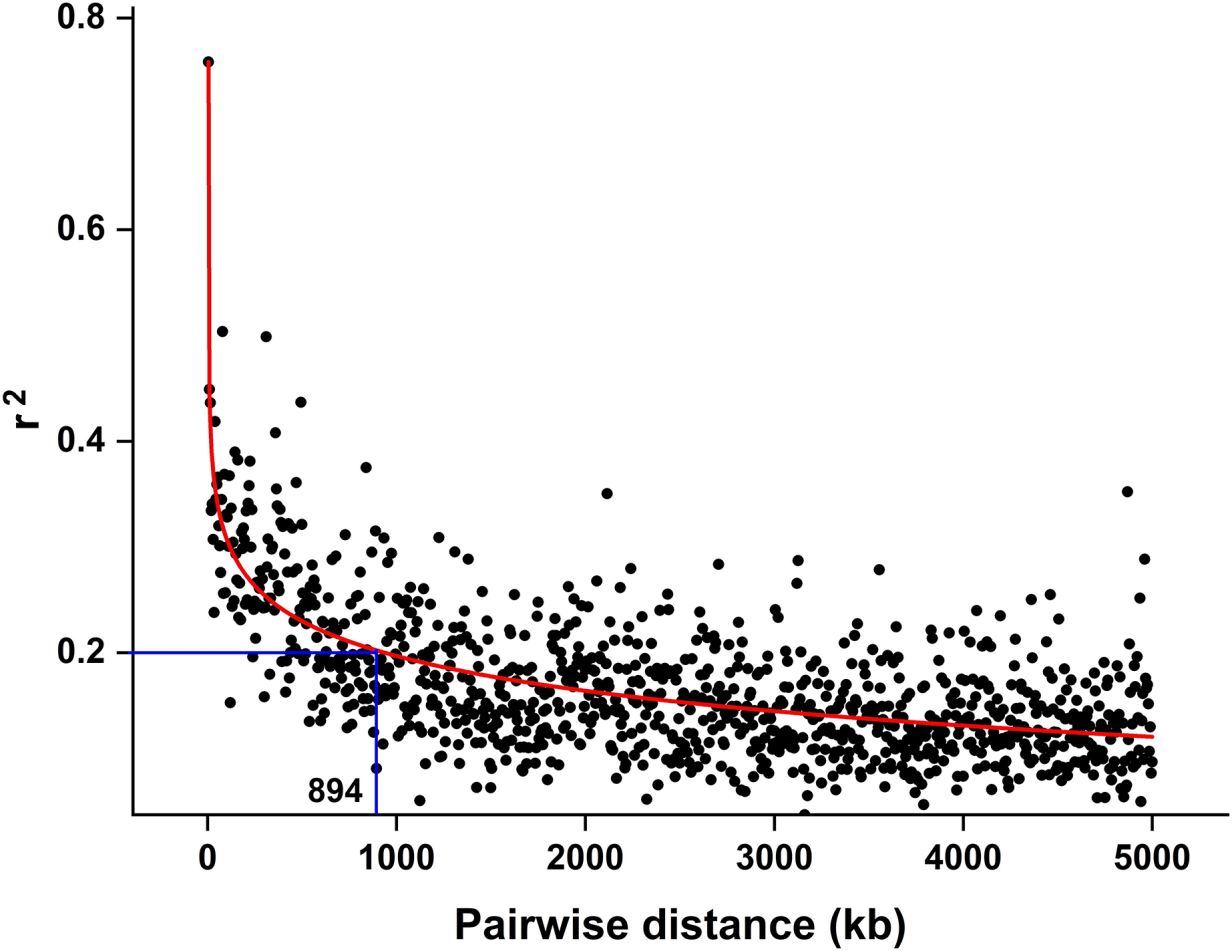
LD decay estimated by the squared allele frequency correlation (*r^2^*) against the pairwise distance between 3,839 molecular markers used in this study. The black scatter points represent the average *r^2^* value of each distance segment, the red curve is the fitting of the scatter points by the OriginPro 2021b software and the blue lines represent an *r^2^* cutoff of 0.2 that was chosen to define the extent of LD in the population.

The CMplot package of R (https://github.com/YinLiLin/CMplot) was used to plot our 3,839 markers and the distribution of known BaYMV disease resistance genes on chromosomes (Fig. 2). The LD analysis was analyzed as the factor that influences the power of detecting a QTL (Brito et al., 2011; Mucha, Bunger & Conington, 2015). The LD between pairs of SNPs was calculated as the squared allele frequency correlation (*r*^*2*^) using TASSEL 5.2 software (Bradbury et al., 2007) as described previously (Megerssa et al., 2021). The LD plot was drawn by OriginPro 2021b software (https://www.originlab.com/). In this study, when *r*^*2*^ = 0.2, the LD of the association panel was approximately 894 kb (Fig. 3).

### GWAS for BaYMV disease resistance

GWAS was performed using the rMVP package in R (Yin et al., 2021). Based on the observation of *p*-values in different models under quantile–quantile (Q–Q) plots, the FarmCPU was selected as the most suitable model (Kaler et al., 2017; Zhang et al., 2021). The Bonferroni of false discovery rate (FDR) correction of 5% calculated by the rMVP package was used for multiple comparison adjustment and as a threshold to identify significant marker-trait associations (Aguado et al., 2020; Megerssa et al., 2021).

In this study, the Bonferroni of FDR correction of the natural population was approximately 4.88. Markers at *p* ≤ 10^−4.88^ (−log10(*p*) ≥ 4.88) level detected by GWAS were defined as significant marker-trait associations (MTAs).

The phenotypic variance explained (PVE) by MTA was calculated using the following formula:

${\rm PVE} = \displaystyle{{{V_Q}} \over {{V_P}}} \times 100{\rm \% }$

${V_Q} = 4{f_{REF}}{f_{ALT}}{\left[ {\displaystyle{1 \over 2}\left( {{\mu _{REF}} - {\mu _{ALT}}} \right)} \right]^2}$

In the formula, ${V_Q}$ represents the genotypic variance, ${V_P}$ represents the phenotypic variance, ${f_{REF}}$ and ${f_{ALT}}$ represent the frequency of the two genotypes of markers, respectively, and ${\mu _{REF}}$ and ${\mu _{ALT}}$ represent the average phenotypic values corresponding to the two genotypes (Wang, 2014).

The genes between the LD upstream and the LD downstream of the MTAs were therefore functionally annotated based on barley Morex V3 reference genome annotation (https://apex.ipk-gatersleben.de/apex/f?p=284:57).

### Sequence analysis of BaYMV

**Figure 4:**
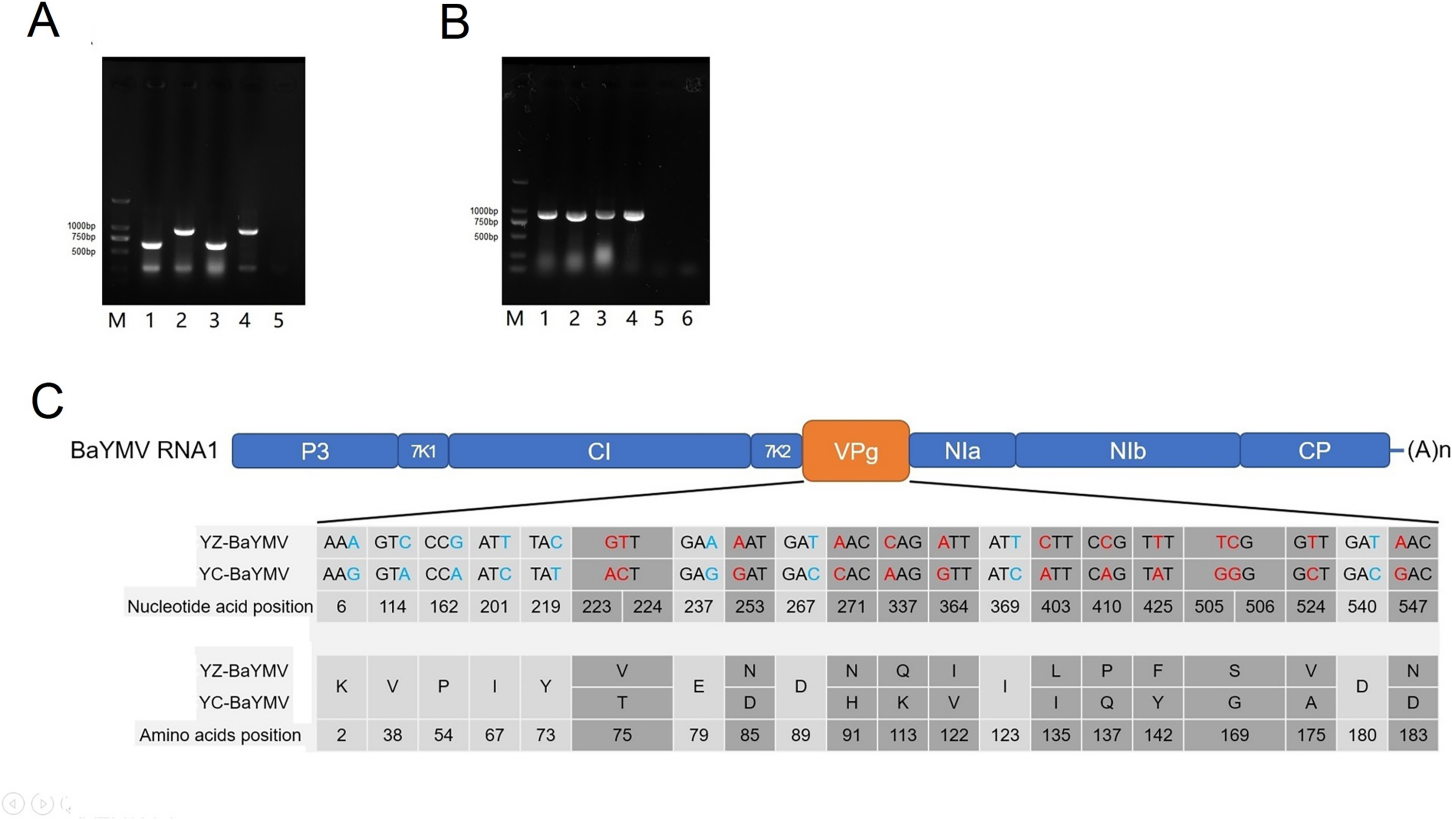
Sequence analysis of BaYMV. (A) Identification of virus species by a multiplex RT-PCR with BaYMV- and BaMMV-specific primer sets M: 2000bp DNA marker (Takara Bio, Beijing, China); 1: Yangzhou Supi 1; 2: Yangzhou Dan 2; 3: Yancheng Supi 1; 4: Yancheng Dan 2; 5: amplifying with H_2_O. (B) The VPg coding sequences of different virus strains amplified from two susceptible barley accessions. M: 2000bp DNA marker (Takara Bio, Beijing, China) 1: amplifying BaYMV VPg from Yangzhou Supi 1; 2: amplifying BaMMV VPg from Yangzhou Dan 2; 3: amplifying BaYMV VPg from Yancheng Supi 1; 4: amplifying BaMMV VPg from Yancheng Dan 2; 5: Negative control, amplifying BaYMV VPg primer with H_2_O; 6: Negative control, amplifying BaMMV VPg primer with H_2_O. (C) Differences of BaYMV-VPg coding sequence between the Yangzhou testing site and the Yancheng testing site. The blue SNP is the synonymous mutation and the red SNP is the non-synonymous mutation.

At the height of the BaYMV disease period in Yangzhou and Yancheng, the infected leaves of susceptible barley accessions ‘Supi 1’ and ‘Dan 2’ were collected for RNA extraction. Total RNA was extracted by RNAiso plus (Takara Bio, Beijing, China) before the general quality and concentration were determined *via* the use of an OD-1000+ spectrophotometer™ (One Drop, Shanghai, China). Total cDNA was obtained by reverse transcription using a 1st strand cDNA synthesis kit (Takara Bio, Beijing, China). A total of 50–60 ng of 1st strand cDNA was input as the template to a 20 μL multi-RT-PCR reaction for identification of BaYMV in leaves displaying disease symptoms (Zou, Wang & Xu, 2016; Shi et al., 2019). The 277 bp and 648 bp fragments were detected in ‘Supi 1’, corresponding to the coding sequence of *HvACTIN* and BaYMV specific fragments, respectively. The 277 bp and 940 bp fragments were detected by multi-RT-PCR in ‘Dan 2’, corresponding to the coding sequence of *HvACTIN* and BaMMV-specific fragments, respectively (Fig. 4A). The results showed that ‘Supi 1’ was only infected with BaYMV and ‘Dan 2’ was only infected with BaMMV at the two assessed sites.

BaYMV and BaMMV terminal binding protein VPg encoding sequences were amplified by specific primers (Table S5) following a procedure similar to previously reported (Jiang et al., 2022). Targeted fragments were verified by Sanger sequencing (Beijing Genomics Institute, Beijing, China). The sequencing data were assembled using MEGA X software (Kumar et al., 2018) and saved as a FASTA file (Table S6).

## Results

### Phenotypic variation of BaYMV disease

**Figure 5:**
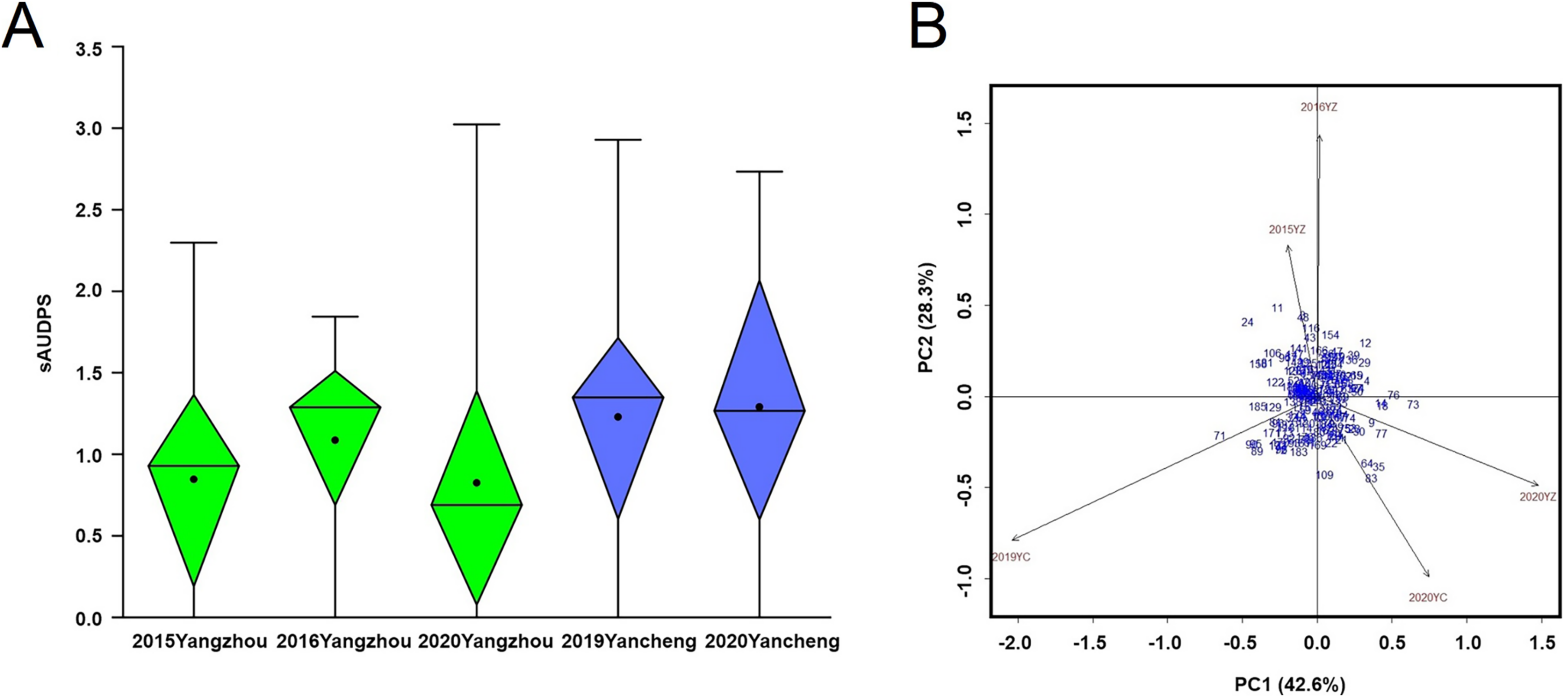
BaYMV disease phenotype of 181 barley accessions. (A) Standardized area under the disease progress steps (sAUDPS) index at 3 years (2015, 2016, and 2020) in the Yangzhou testing site and 2 years (2019 and 2020) in the Yancheng testing site. (B) AMMI biplot for the sAUDPS indexes in different environments. Blue numbers represent accessions. Brown numbers represent the stability of the environment.

The average incidence of BaYMV disease indicated that the mildest outbreak occurred in Yangzhou in 2020 while the most severe outbreak occurred in Yancheng in 2019 (Fig. 5A). The sAUDPS indexes were significantly positively correlated (*p* < 0.01) in all environments (Table 1).

**Table 1:** Correlations analysis of sAUDPS indexes in different environments.

Environment	Mean	Range	CV (%)	Correlation coefficient				
				2015 Yangzhou	2016 Yangzhou	2020 Yangzhou	2019 Yancheng	2020 Yancheng
2015 Yangzhou	0.85	0.00–2.30	74.14					
2016 Yangzhou	1.09	0.00–1.84	51.93	0.86** *P* = 2.8384E−53				
2020 Yangzhou	0.83	0.00–3.02	97.54	0.74** *P* = 5.8099E−33	0.65** *P* = 2.2727E−23			
2019 Yancheng	1.23	0.00–2.93	59.73	0.76** *P* = 2.1563E−35	0.71** *P* = 2.7978E−29	0.62** *P* = 2.9198E−20		
2020 Yancheng	1.29	0.00–2.73	62.19	0.80** *P* = 8.0229E−41	0.72** *P* = 4.9833E−30	0.68** *P* = 1.0375E−25	0.82** *P* = 4.0418E−45	

**Note:**

** Represent significant correlation at 0.01 levels (*p* < 0.01).

In the analysis of variance, accessions and sites were considered fixed and significant differences (*p* < 0.01) were found between accessions and sites for all dependent variables (Table 2). These results indicated that the incidence of BaYMV disease was not only affected by genetic factors, but also by environmental factors, the year of analysis and even ecological conditions from the testing sites.

**Table 2:** Analysis of variance (ANOVA) for sAUDPS indexes for 181 barley accessions evaluated under two testing sites.

SOV	df	Sum of squares	Mean square	*F*-value	*P*-value
Accessions	180	1,029.62	5.72	42.73**	0.0E0
Sites	4	86.12	21.53	160.90**	6.3327E−118
Accessions × Sites	740	338.54	0.47	3.51**	1.7228E−102
Error	1,860	242.20	0.13		

**Notes:**

df: degree of freedom.

** Represent significance at 0.01 levels (*p* < 0.01).

### Stability analysis of resistance to BaYMV disease

PCA was used to perform the interaction analysis between phenotype and environment. The result showed that there were significant differences (*p* < 0.01) among the values of Interaction Principal Components Axes 1, 2, and 3 (IPCA1, IPCA2, and IPCA3) (Table S1; Table 3), indicating that there is a significant interaction between the sAUDPS index and environment. The accumulative contribution rate of the three IPCA was 92.60%.

**Table 3:** Principal component analysis (PCA) of sAUDPS indexes of all testing sites.

SOV	Contributing rate (%)	df	Sum of Squares	Mean Square	*F*-value	*P*-value
IPCA1	42.60	183	144.33	0.79	6.02**	0.00E+00
IPCA2	28.30	181	95.88	0.53	4.04**	0.00E+00
IPCA3	21.60	179	73.13	0.41	3.12**	0.00E+00
IPCA4	7.40	177	25.20	0.14	1.09	0.2051

**Notes:**

df: degree of freedom.

** Represent significance at 0.01 levels (*p* < 0.01).

According to the significant principal component score, the stability parameter *D value* of the sAUDPS index of each accession was calculated and sorted in ascending order. The *D value* was expressed on the AMMI biplot by the distance between the accession to the origin (Fig. 5B). Among the top ten accessions, seven showed stable resistance to BaYMV disease, all having a lower sAUDPS index in different sites and years (Table 4).

**Table 4:** Top ten barley accessions with phenotypic stability in all environments and their sAUPDS indexes.

Ranking	Variety	*D* value	Yangzhou			Yancheng		Average
			2015	2016	2020	2019	2020	
1	Qimendamai	0.0131	0.95	1.41	1.13	1.40	1.60	1.30
2	Yangnongpi 7	0.0195	0.06	0.31	0.00	0.34	0.40	0.22
3	Yangnongpi 8	0.0248	0.19	0.36	0.13	0.60	0.47	0.31
4	Zhe 35-21	0.0248	0.00	0.13	0.00	0.34	0.33	0.16
5	Yanyin 1	0.0265	0.49	1.27	0.87	1.68	1.20	0.99
6	Yang 95168 × Yan 94130	0.0411	0.01	0.04	0.00	0.24	0.20	0.10
7	Changmangluodamai	0.0416	1.16	1.49	1.34	1.42	1.67	1.42
8	Liudanzhun	0.0427	0.00	0.02	0.00	0.31	0.33	0.09
9	Yan 99175	0.0491	0.08	0.22	0.06	0.18	0.47	0.19
10	Sunong 16	0.0492	0.16	0.49	0.00	0.53	0.47	0.33

### Marker-trait associations

**Figure 6:**
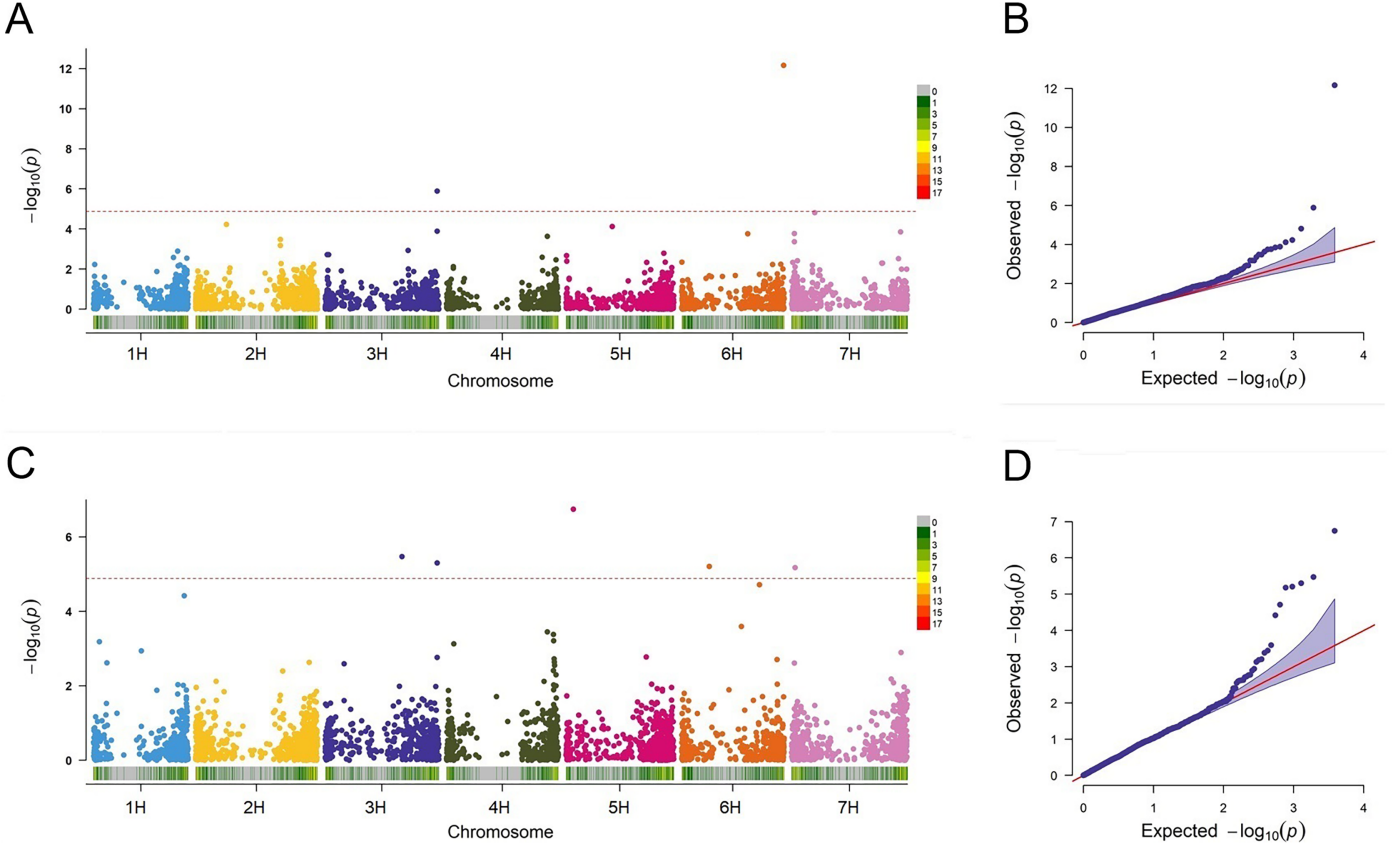
Association analysis for BaYMV disease resistance in the Yangzhou testing site and the Yancheng testing site by FarmCPU model. (A/C) Manhattan maps show marker-trait associations in the Yangzhou and Yancheng testing sites, respectively. The abscissa shows the physical location of the SNPs, and the SNPs on different chromosomes were distinguished by color. SNP density was expressed in a manner corresponding to the abscissa. (B/D) Quantile–Quantile Plots of genome-wide association analysis in the Yangzhou and Yancheng testing sites, respectively.

In this study, the FarmCPU approach was employed to detect the associations between the markers and BaYMV disease resistance. QLB1 (*p* = 1.29E−06) and SNP2571 (*p* = 6.94E−13) associated with the disease in Yangzhou were detected on chromosomes 3H at 612 Mbp and 6H at 557 Mbp, respectively (Fig. 6A). Among these MTAs, QLB1 explained 28.58% variation and SNP2571 explained 24.21% variation at the Yangzhou site, indicating a high cluster of mapped genes and a novel resistance locus, respectively (Table 5).

**Table 5:** Significant (the Bonferroni of false discovery rate (FDR) < 0.05) single nucleotide polymorphisms (SNPs) associated with BaYMV disease resistance identified using FarmCPU model.

Site	Marker	Chr	POS/bp	REF	ALT	*p* value	PVE (%)	Estimate of marker
Yangzhou	QLB1	3H	612473728	G	T	1.29E−06	28.58	Mapped gene
	SNP2571	6H	557771117	T	C	6.94E−13	24.21	Novel resistant locus
Yancheng	SNP3426	3H	419648980	C	T	3.36E−06	0.28	
	QLB1	3H	612473728	G	T	5.03E−06	31.80	Mapped gene
	SNP4036	5H	40265162	A	G	1.81E−07	19.23	Novel resistant locus
	SNP1839	6H	153073031	C	T	6.21E−06	2.74	
	SNP0024	7H	18589900	A	G	6.66E−06	19.79	Novel resistant locus

A total of five MTAs were detected in Yancheng. Three highly significant ones, QLB1 (*p* = 5.03E−06), SNP4036 (*p* = 1.81E−07), and SNP0024 (*p* = 6.66E−06) had PVE values ranging from 19.23% to 31.80% and they are located on chromosomes 3H at 612 Mbp, 5H at 40 Mbp and 7H at 18 Mbp, respectively. Two less significant MTAs, SNP3426 (*p* = 3.36E−06) and SNP1839 (*p* = 6.21E−06), had PVE values ranging from only 0.28% to 2.74% (Fig. 6C, Table 5).

### Sequence analysis of VPg

The BaYMV VPg coding sequence (880 bp fragment) was amplified using ‘Supi 1’ collected from both sites, and the BaMMV VPg coding sequence (830 bp fragment) was amplified from ‘Dan 2’ collected from both sites (Fig. 4B). After sequencing, multi-sequence alignment was performed using the MUSCLE program in MEGA X software. There were twenty-two SNP differences in the coding sequence of YZ-BaYMV and YC-BaYMV, among which thirteen non-synonymous mutations resulted in eleven amino acid changes (Fig. 4C). The rate of amino acid mutation between two VPg coding sequences was 5.34% (11/206). There was no SNP difference between YZ-BaMMV and YC-BaMMV.

## Discussion

### Detection of known resistance genes to BaYMV disease

In this study, markers associated with known BaYMV disease resistance genes were used for genotyping the natural population, and twelve markers showed significant associations (*p* < 0.01) with disease resistance (Table S2). Some known disease resistance genes derived from a single accession are rarely used in disease resistance breeding (Le Gouis et al., 2004; Kai et al., 2012; Johnston et al., 2015; Pidon et al., 2020). The MTA detected in multi-environments was QLB1, which was the diagnostic SSR marker for *rym4*/*rym5* (Tyrka et al., 2008). In this study, the QLB1 showed the closest association with BaYMV disease resistance (Table S2), indicated by the highest PVE (31.80%) (Table 5). Therefore, the known BaYMV disease resistance genes *rym4/rym5* are still the major resistance genes in the natural population used in this study.

### Discovery of novel resistance loci to BaYMV disease

In this study, the FarmCPU was used to detect the associations between the molecular markers and BaYMV disease resistance under different BaYMV/BaMMV-infected nurseries. In Yangzhou, an MTA (SNP2571) which explained 24.21% of phenotypic variation was identified at the terminal region of chromosome 6H (Figure 6A), different from the known BaYMV disease resistance genes *Rym14*^*Hb*^ (Pidon et al., 2020) and *rym15* (Le Gouis et al., 2004) which are mapped at the other terminal region of chromosome 6H (Fig. 2). Based on the LD decay distance, the genes between the 894 kb upstream and 894 kb downstream of SNP2571 were defined as candidate genes. According to the annotation information of the Morex V3 reference genome, a total of 14 typical plant disease resistance genes (*R* genes) were enriched in this region (Table S7). Among them, two genes encoding leucine-rich repeat proteins (*HORVU.MOREX.r3.6HG0631890* and *HORVU.MOREX.r3.6HG0632320*), five genes encoding NBS-LRR disease resistance protein-like proteins (*HORVU.MOREX.r3.6HG0631950*, *HORVU.MOREX.r3.6HG0632390*, *HORVU.MOREX.r3.6HG0632400*, *HORVU.MOREX.r3.6HG0632430* and *HORVU.MOREX.r3.6HG0632630*) and seven genes encoding homologs to the disease resistance protein RPM1 (*HORVU.MOREX.r3.6HG0632440*, *HORVU.MOREX.r3.6HG0632480*, *HORVU.MOREX.r3.6HG0632490*, *HORVU.MOREX.r3.6HG0632530*, *HORVU.MOREX.r3.6HG0632550*, *HORVU.MOREX.r3.6HG0632610* and *HORVU.MOREX.r3.6HG0632650*) putatively represent the candidates genes for the novel BaMYV disease resistance QTL on chromosome 6H.

At present, only one BaYMV disease resistance gene, *rym3/Undesigned* has been identified on chromosome 5H in barley, which was mapped within a 174 Mbp range at a position 362–536 Mbp on the chromosome (Saeki et al., 1999; Werner et al., 2003b). In this study, one new MTA (SNP4036) was detected at the physical position of 40 Mbp on chromosome 5H (Table 5), which could explain 19.23% of phenotypic variation in Yancheng. Based on the LD decay (894 kb) of the association panel, a total of 23 annotated barley genes were identified for the disease resistance QTL (Table S7).

*rym2* and *rym7t* mapped at the interval position from 213 Mbp to 369 Mbp on chromosome 7H showed resistance to BaYMV disease (Gotz & Friedt, 1993; Takata et al., 2012). In this study, SNP0024 at a position 18 Mb from the start of chromosome 7H was significantly associated with BaYMV disease. This novel and significant MTA explained 19.79% of phenotypic variation in Yancheng (Table 5). There were 81 annotated barley genes in the confidence interval of the novel resistance QTL near SNP0024, of which two genes encoding NBS-LRR disease resistance like proteins (*HORVU.MOREX.r3.7HG0644180* and *HORVU.MOREX.r3.7HG0644290*) and another two genes encoding homologs of the disease resistance protein RPM1 (*HORVU.MOREX.r3.7HG0644780* and *HORVU.MOREX.r3.7HG0644810*) were potential candidate genes for the novel disease resistance QTL on chromosome 7H (Table S7).

### Differences of BaYMV disease in different nurseries

There have been many reported disease resistance genes that maintain variable levels of resistance to diverse BaYMV viral strains (Zheng et al., 1999; Jiang et al., 2020). In this study, variance analysis showed that there were significant differences (*p* < 0.01) in the natural population of BaYMV disease between these two sites. The mean sAUDPS index in Yangzhou from 3 years was lower than that in Yancheng from 2 years, indicating that the difference observed was due to differences in viral pathogenicity. Moreover, each testing site had independent resistance loci, except for QLB1 which was detected at both sites. Previous studies have shown that there were differences between strains of BaYMV in Yangzhou and Yancheng (Chen et al., 1999). Our results showed eleven amino acid differences in the BaYMV-VPg coding sequence between Yangzhou and Yancheng. VPg is a multifunctional protein, which directly participates in almost every key process of virus infection *via* the formation of different splice forms. VPg especially functions in protein maturation and virus movement during the process of infection, thus fundamentally determines viral pathogenicity (Stein et al., 2005; Charron et al., 2008; Li et al., 2016). Therefore, the difference in the VPg coding sequence of the BaYMV virus might be the reason for the difference in the severity of the BaYMV disease observed across the two testing sites.

### Prospects of BaYMV disease resistance breeding

Under the natural environment, the dormant spores of *Polymyxa graminis* carrying virus particles exist in the soil and diseased root residues. Cultivation measures such as applying sterilizing agents, crop rotation and postponing the sowing date may alleviate the effect of BaYMV disease but these measures are not ecologically nor environmentally friendly or sustainable. In this way, the most efficient and environment-friendly way to alleviate BaYMV infection is to cultivate varieties of resistant species.

The relationship between virus and host plant disease resistance follows a typical pattern of gene-gene interaction. Although there are many identified genes regulating crop resistance to BaYMV disease, however, due to the rapid evolution of the virus, the positive effects from these genes are becoming increasingly diminished. Identifying novel genes conferring enhanced crop resistance to the evolving BaYMV is thus very necessary (Yang et al., 2014a; Yang et al., 2017) for offering new genetic resources for breeding accessions of barley resistant to BaMYV disease (Yang et al., 2017; Jiang et al., 2020).

In this study, the MTA (SNP2571) detected on chromosome 6H consisted of two alleles (C-allele and T-allele). A *t*-test conducted on the phenotype between the two groups at the Yangzhou site showed that the disease symptoms of the accessions containing the C-allele were significantly (*p* < 0.01) lower than those of the accessions containing the T-allele. In our study, the disease resistance allele of SNP2571 was mostly found in six-rowed landraces (*n* = 59, 32.60%). Similarly, the resistance allele of the MTA on chromosome 7H was also mostly discovered in six-rowed landraces (*n* = 44, 24.31%) (Table S8), supporting previous reports that novel genetic resources of barley resistant to BaMYV disease derived from landraces (Shi et al., 2019; Jiang et al., 2020) could be used in plant breeding for disease resistance. It should be emphasized that many *R* genes were enriched in the candidate interval between SNP2571 and SNP0024 based on LD analysis (Table S7).

## Conclusions

Breeding disease resistance crop accessions is the most effective, sustainable and environmentally friendly approach to alleviate the loss of yield stemming from BaYMV disease. This study showed the value of utilizing AMMI as the parameter to screen barley accessions that harbor stable resistance to BaYMV disease. Several BaYMV disease resistance loci were identified utilizing a combination of the GWAS technique and the sAUDPS method for the first time. The known BaYMV disease resistance genes, *rym4* and *rym5*, still provide barley with a certain degree of disease resistance. The landraces which carry favorable alleles for BaYMV disease resistance can be used for breeding new elite accessions of barley which are resistant to this disease in the future.

## Supplemental Information

10.7717/peerj.13128/supp-1 Supplemental Information 1 Name, characterization, geographical source, and detailed phenotypic information of the 181 barley accessions.

10.7717/peerj.13128/supp-2 Supplemental Information 2 Information of markers associated with 22 known barley yellow mosaic disease-resistant genes and association analysis in the 181 barley accessions. The physical positions of markers associated to rym3/Undesigned had large differences. According to the references, the gene is located in chromosome 5HS, so the physical positions of cMWG770, MWG526 and MWG596 are used as candidate intervals.
The red font indicates that the correlation is significant at the 0.01 level (2-tailed).

10.7717/peerj.13128/supp-3 Supplemental Information 3 Distribution of markers used in GWAS analysis on barley chromosomes.

10.7717/peerj.13128/supp-4 Supplemental Information 4 Raw genotype data used in GWAS analysis (including genotype in VCF format, kinship, and structure matrix).

10.7717/peerj.13128/supp-5 Supplemental Information 5 Sequence information and purposes of PCR primers used in this study.

10.7717/peerj.13128/supp-6 Supplemental Information 6 Sequencing data of BaYMV and BaMMV terminal binding protein VPg encoding sequence.

10.7717/peerj.13128/supp-7 Supplemental Information 7 Candidate genes annotated in the confidence intervals of three novel BaYMV disease resistance loci. Green highlights represent plant disease resistance genes. Yellow highlights represent significant association loci.

10.7717/peerj.13128/supp-8 Supplemental Information 8 Distribution of disease resistance alleles at three novel loci the 181 barley accessions.

## Additional Information and Declarations

### DNA Deposition

The following information was supplied regarding the deposition of DNA sequences: The VPg sequences of two Barley yellow mosaic viruses in two nurseries are available at GenBank: OK335779 and OK335780. Detailed sequence information is available in Table S6.

### Data Availability

The following information was supplied regarding data availability: The material information and phenotype data of the plant material, raw genotype data of the plant material and the VPG sequences of two viruses in two disease nurseries are available in the Supplemental Files.
